# Evaluation of a 2D UNet-Based Attenuation Correction Methodology for PET/MR Brain Studies

**DOI:** 10.1007/s10278-021-00551-1

**Published:** 2022-01-28

**Authors:** Luca Presotto, Valentino Bettinardi, Matteo Bagnalasta, Paola Scifo, Annarita Savi, Emilia Giovanna Vanoli, Federico Fallanca, Maria Picchio, Daniela Perani, Luigi Gianolli, Elisabetta De Bernardi

**Affiliations:** 1 Nuclear Medicine Department, IRCCS San Raffaele Scientific Institute, Milan, Italy; 2 Vita-Salute San Raffaele University, Milan, Italy; 3 School of Medicine and Surgery, University of Milano-Bicocca, via Cadore 48, Monza, 20900 Italy; 4 Bicocca Bioinformatics Biostatistics and Bioimaging Centre - B4, University of Milan-Bicocca, Monza, Italy

**Keywords:** PET/MR, Brain attenuation correction, Deep learning, UNet

## Abstract

Deep learning (DL) strategies applied to magnetic resonance (MR) images in positron emission tomography (PET)/MR can provide synthetic attenuation correction (AC) maps, and consequently PET images, more accurate than segmentation or atlas-registration strategies. As first objective, we aim to investigate the best MR image to be used and the best point of the AC pipeline to insert the synthetic map in. Sixteen patients underwent a 18F-fluorodeoxyglucose (FDG) PET/computed tomography (CT) and a PET/MR brain study in the same day. PET/CT images were reconstructed with attenuation maps obtained: (1) from CT (reference), (2) from MR with an atlas-based and a segmentation-based method and (3) with a 2D UNet trained on MR image/attenuation map pairs. As for MR, T1-weighted and Zero Time Echo (ZTE) images were considered; as for attenuation maps, CTs and 511 keV low-resolution attenuation maps were assessed. As second objective, we assessed the ability of DL strategies to provide proper AC maps in presence of cranial anatomy alterations due to surgery. Three 11C-methionine (METH) PET/MR studies were considered. PET images were reconstructed with attenuation maps obtained: (1) from diagnostic coregistered CT (reference), (2) from MR with an atlas-based and a segmentation-based method and (3) with 2D UNets trained on the sixteen FDG anatomically normal patients. Only UNets taking ZTE images in input were considered. FDG and METH PET images were quantitatively evaluated. As for anatomically normal FDG patients, UNet AC models generally provide an uptake estimate with lower bias than atlas-based or segmentation-based methods. The intersubject average bias on images corrected with UNet AC maps is always smaller than 1.5%, except for AC maps generated on too coarse grids. The intersubject bias variability is the lowest (always lower than 2%) for UNet AC maps coming from ZTE images, larger for other methods. UNet models working on MR ZTE images and generating synthetic CT or 511 keV low-resolution attenuation maps therefore provide the best results in terms of both accuracy and variability. As for METH anatomically altered patients, DL properly reconstructs anatomical alterations. Quantitative results on PET images confirm those found on anatomically normal FDG patients.

## Introduction

Several investigators have shown that synthetic computed tomography (CT) images can be obtained with deep learning (DL) strategies applied to magnetic resonance (MR) images [1–7]. This approach currently appears to be the most promising both in attenuation correction (AC) of positron emission tomography (PET) images in hybrid PET/MR scanners and in MR-based radiotherapy treatment planning [8, 9]. In this work, we focused on the AC of brain PET/MR studies, where the challenge is to estimate 511 keV gamma ray attenuation maps to correctly reconstruct the uptake of an injected radiotracer in brain areas.

The AC pipeline implemented in PET/MR scanners directly derives from PET/CT scanner AC pipelines and schematically consists of the following steps: (1) generation of a synthetic CT image corresponding to a high resolution map of tissue attenuation coefficients at ~80–120 keV; (2) conversion from ~80 to 120 keV attenuation coefficients to 511 keV attenuation coefficients by means of bilinear or trilinear functions; (3) smoothing with Gaussian kernel to match PET low spatial resolution; (4) resampling on a coarser grid (for some vendors). The obtained 511 keV low-resolution attenuation map (LRAM) is then used inside the PET image reconstruction process. Tools currently implemented on PET/MR scanners generate synthetic CT images following atlas-based or segmentation-based methods. In atlas-based approaches, single or multiple pairs of MR/CT templates are registered to patient MR images; in segmentation-based approaches, MR images are segmented in three or four tissue classes, and proper attenuation coefficients are then assigned to voxels in each class [10]. In brain studies, Ultrashort Echo Time (UTE) or Zero Echo Time (ZTE) MR sequences are generally used, thanks to their ability to collect MR signal from bones [11].

Most of the DL approaches presented so far in literature for AC correction in PET/MR brain studies aim to construct synthetic CT images starting from diagnostic [12, 13] or non-diagnostic [4, 14, 15] MR images. These methods outperform conventional synthetic CT construction methods, reducing the average bias in PET quantification from about 5% to about 2% [8, 9]. The rationale at the base of this work is that synthetic CTs, which are not used for diagnostic purpose, are not needed for AC in PET/MR studies if synthetic LRAM images can be directly generated. LRAMs contain information at lower resolution and contrast than CTs, and therefore, simpler and leaner DL networks than those required to generate CTs may be sufficient. An approach of this kind has been followed by Spuhler et al. [3] who used a Convolutional Neural Network (CNN) to directly estimate LRAMs, starting from MR T1-weighted (T1) images. T1 images may however be suboptimal for X-ray attenuation estimation, since they do not collect any signal from air and cortical bone, which both appear comparably dark. ZTE images are expected to perform better than conventional sequences in this task, thanks to their ability to discriminate air and bone and to the inversely proportional relationship between ZTE signal intensity and attenuation coefficients in bones [16].

The aim of this work is therefore to generate LRAMs for AC in brain PET/MR from ZTE images and to compare their AC performance against T1-based LRAMs and T1- or ZTE-based synthetic CTs. A unique standard 2D UNet architecture [17] was used throughout the study. Synthetic images to be used at different points of the AC pipeline were generated, namely synthetic CTs, synthetic LRAMs before resampling and synthetic LRAMs after resampling. T1-weighted images or ZTE images were considered as inputs. AC-corrected reconstructed PET images were compared, taking as reference the ones obtained with patient-specific true CT images. The proposed methodologies were assessed both on normal and surgically altered cranial anatomies. Atlas-based and segmentation-based methods were also considered for comparison.

## Material and Methods

### Brief Literature Review

MR-based DL approaches presented so far in literature for AC correction in brain PET/MR are described in Table 1.

**Table 1:** MR-based DL approaches for AC correction in brain PET/MR

**Ref**	**Input**	**Output**	**Network**	**No. in train and test**	**Dice in bone regions**	**Regional PET bias**	**PET surface error**	**Anatomic abnormalities**
Gong et al. 2018 [14]	Dixon	pseudoCT	2.5D UNet	40 (cross validation)	0.76	< 3% (8 VOIs)	No	No
	Dixon + ZTE	pseudoCT	2.5D GroupUNet	14 (cross validation)	0.80	< 3% (8 VOIs)	No	No
Blanc-Durand et al. 2019 [4]	ZTE	PseudoCT	3D UNet	Train 23 Test 47	No	< 2% (70 VOIs)	No	No
Arabi et al. 2019 [12]	T1	PseudoCT	3D DL-AdvSS	40 (cross validation)	0.80	< 3.5% (63 VOIs)	No	No
Spuhler et al. 2019 [3]	T1	LRAM	2D UNet	Train 55 Test 11	No	< 3% (19 VOIs)	No	No
Tao et al. 2021 [13]	T1	pseudoCT	2D cGAN	11 (cross validation)	No	No	No	No
	Dixon	pseudoCT	2D cGAN	10 (cross validation)	No	No	No	No
Gong et al. 2021 [15]	T1	pseudoCT	2.5D GroupUNet	35 (cross validation)	0.84	< 2% (10 VOIs)	Yes	No
	Dixon	pseudoCT	2.5D GroupUNet	35 (cross validation)	0.84	< 2% (10 VOIs)	Yes	No
	mUTE	pseudoCT	2.5D GroupUNet	35 (cross validation)	0.87	< 2% (10 VOIs)	Yes	No

### Patient Information

Two datasets were considered in this study. The first one (indicated as FDG dataset in the following) contains images of sixteen patients (9 men, 7 women, 68 ± 9 years old) who underwent a 18F-FDG PET/CT study (Discovery-STE scanner, General Electric Medical Systems, GEMS, Waukeska, WI, USA) for a neurological evaluation of cognitive impairment and accepted to perform a second scan on a PET/MR system (PET/MR SIGNA, GEMS, Waukeska, WI, USA). The double-study protocol was approved by the IRCCS San Raffaele Hospital local ethical committee and patients signed a written informed consent form. The second dataset (indicated as METH dataset in the following) contains images of three women who underwent a 11C-methionine-PET/MR study for oncological evaluation after radiotherapy. These patients were selected to test the proposed techniques as they present cranial anatomy variations due to surgery. They all signed the informed consent allowing using images for research and educational purpose.

### FDG PET/CT Data Acquisition

A standard 18F-FDG PET imaging procedure was performed [18]. Patient preparation required at least 4 h of fasting, an i.v. injection with 125–250 MBq (average 150 MBq) of 18F-FDG followed by an uptake time of 45 min to achieve an optimal cerebral uptake. Patients were then positioned on the PET/CT scanner bed with the head in a head holder (HH) to reduce head movements. The PET/CT acquisition protocol consisted of a low dose CT scan (120kVp, 30 mA) followed by a 3D PET study (15 min).

### FDG PET/MR Data Acquisition

The PET/MR acquisition started at about 90 min from the FDG injection time. The PET/MR study consisted in four MR sequences (3D LAVA-Flex, 3D Proton Density weighted ZTE, T1-weighted 3D-BRAVO and 2D T2-weighted PROPELLER) acquired using a 32-channel coil array within a simultaneous 20-min PET scan. Only the first three MR sequences were used in this work. Acquisition parameters were as follows:

- Axial 3D LAVA-Flex: TR = 4 ms, TE = 1.7 and 2.23 ms, flip angle = 5˚, acquisition matrix = 256 × 128 × 480; reconstructed matrix: 256 × 256, reconstructed FOV = 264 × 264 mm^2^, reconstructed voxel size = 1.95 × 1.95 × 2.6 mm^3^
- 3D ZTE: TR = 399.5 ms, TE = 0.016 ms, flip angle = 0.8˚, acquisition matrix = 110 × 110 × 166, reconstructed matrix = 128 × 128, reconstructed FOV = 264 × 264 mm^2^, reconstructed voxel size = 2.06 × 2.06 × 2.4 mm^3^
- Axial 3D T1-weighted (BRAVO): TR = 8.34 ms, TE = 3.10 ms, TI = 500 ms, flip angle = 12˚, acquisition matrix = 240 × 240 × 328, reconstructed matrix = 512 × 512, reconstructed FOV 240 × 240 mm^2^, reconstructed voxel size 0.47 × 0.47 × 0.5 mm^3^

### 511 KeV LRAM Generation for FDG Patients

FDG PET data acquired with the PET/CT scanner were reconstructed off line with the Recon Research Tool provided by GEMS (PETTOOLBOX 2.0). The reconstruction algorithm requires as inputs PET raw data and a LRAM. In the Discovery-STE PET/CT scanner AC pipeline, the LRAM is obtained by applying a bilinear scaling and a 10-mm full width at half maximum (FWHM) Gaussian filter to CT images and by resampling the result on a coarse voxel grid of 5.47 × 5.47 × 3.27 mm^3^. Eight LRAM volumes were generated:

- LRAM_CT-System_: LRAMs automatically generated by the PET/CT system after processing the original CT images reconstructed on a 0.8 × 0.8 × 2.5 mm^3^ voxel grid. To ensure coherence among multiple datasets, the bilinear scaling function used in the SIGNA PET/MR system was used. PET images reconstructed with LRAM_CT-System_ were considered as reference. LRAMs obtained after the bilinear scaling and the 10 mm FWHM Gaussian filter but before the coarse grid resampling were called LRAM_CT-System-HighRes_ and were used as reference output by UNets generating LRAMs before resampling, as described in the following.
- LRAM_ATLAS_ and LRAM_ZTE_: LRAMs automatically generated by the SIGNA PET/MR system with atlas-based and segmentation-based methods, respectively relying on LAVA-Flex and LAVA-Flex and ZTE sequences. Both LRAM_ATLAS_ and LRAM_ZTE_ accounted for the radiofrequency coil contribution to attenuation. To allow a comparison of the MR based attenuation maps when using PET emission data obtained in the PET/CT study session, the MR coil attenuation component had to be removed and substituted with the PET/CT head holder (HH). Volumes were then registered to CT volumes by means of a rigid transformation with bilinear interpolation using MIPAV software (https://mipav.cit.nih.gov/).
- LRAM_CNN-ZTE_ and LRAM_CNN-T1_: LRAMs obtained with a UNet trained on 0.8 × 0.8 × 2.5 mm^3^ LRAM_CT-System-HighRes_ and ZTE (T1) pairs.
- LRAM_CNN-ZTE-Coarse_: LRAM obtained with a UNet trained on 5.47 × 5.47 × 3.27 mm^3^ LRAM_CT-System_ and ZTE pairs.
- LRAM_CNN-pseudoCT-ZTE_ and LRAM_CNN-pseudoCT-T1_: LRAMs obtained applying the AC pipeline to pseudoCTs (pseudoCT-ZTE and pseudoCT-T1) obtained with a UNet trained on 0.8 × 0.8 × 2.5 mm^3^ CT and ZTE (T1) pairs.

**Fig. 1:**
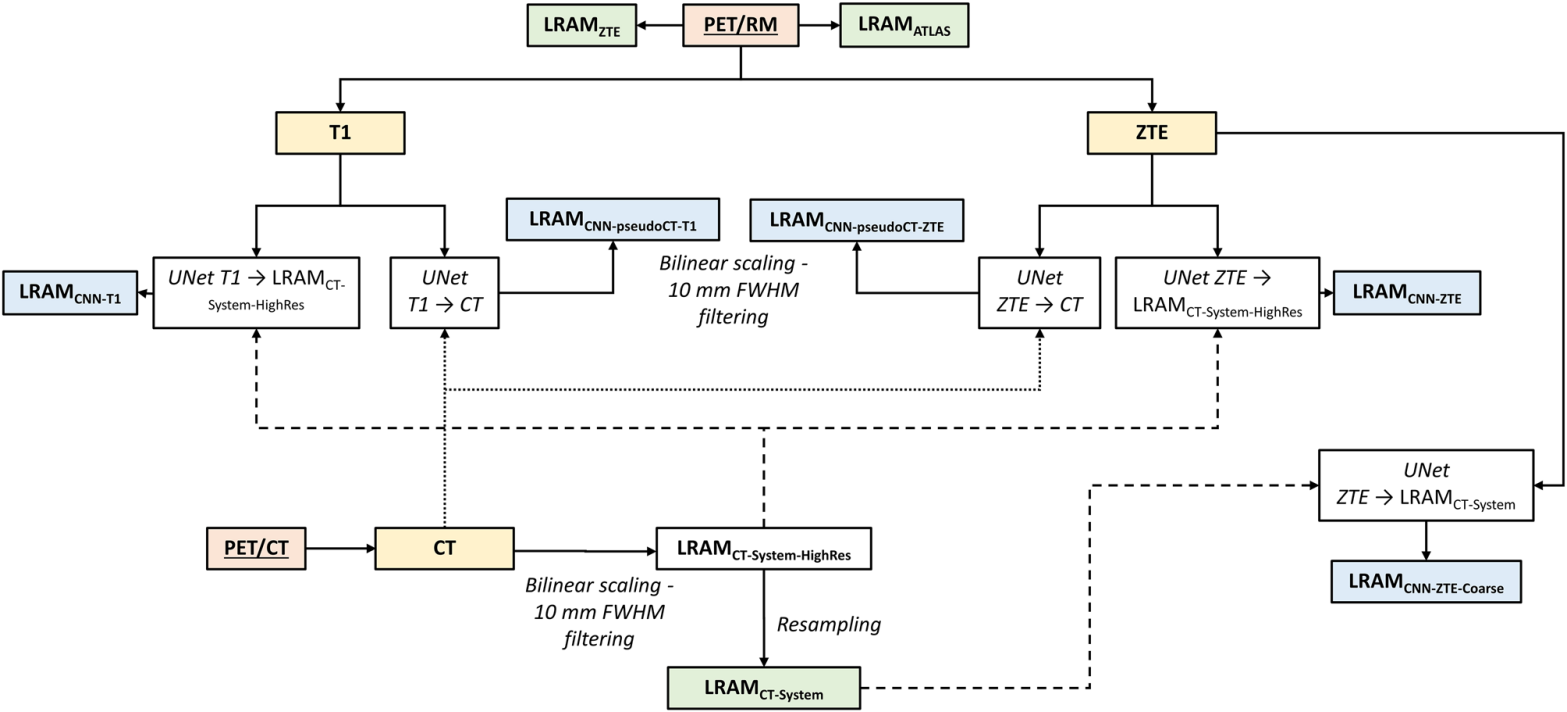
Scheme representing the considered LRAM volumes. In green, LRAMs automatically generated by the PET/CT scanner (LRAM_CT-System_ and LRAM_CT-System–HighRes_, before coarse grid resampling) and by the PET/MR scanner (LRAM_ZTE_ and LRAM_ATLAS_). In blue, LRAMs generated by UNets: (1) LRAM_CNN-T1_ and LRAM_CNN-ZTE_, respectively obtained with UNets trained on T1/LRAM_CT-System-HighRes_ and ZTE/LRAM_CT-System-HighRes_ pairs; (2) LRAM_CNN-pseudoCT-T1_ and LRAM_CNN-pseudoCT-ZTE_, respectively obtained by scaling and filtering pseudoCTs obtained with UNets trained on T1/CT and ZTE/CT pairs; (3) LRAM_CNN-ZTE-Coarse_ obtained with a UNet trained on ZTE/LRAM_CT-System_ pairs. Dotted lines indicate connections used for UNet training. Continuous lines indicate connections used both in UNet training and test

The process of image generation is represented in Fig. 1.

**Fig. 2:**
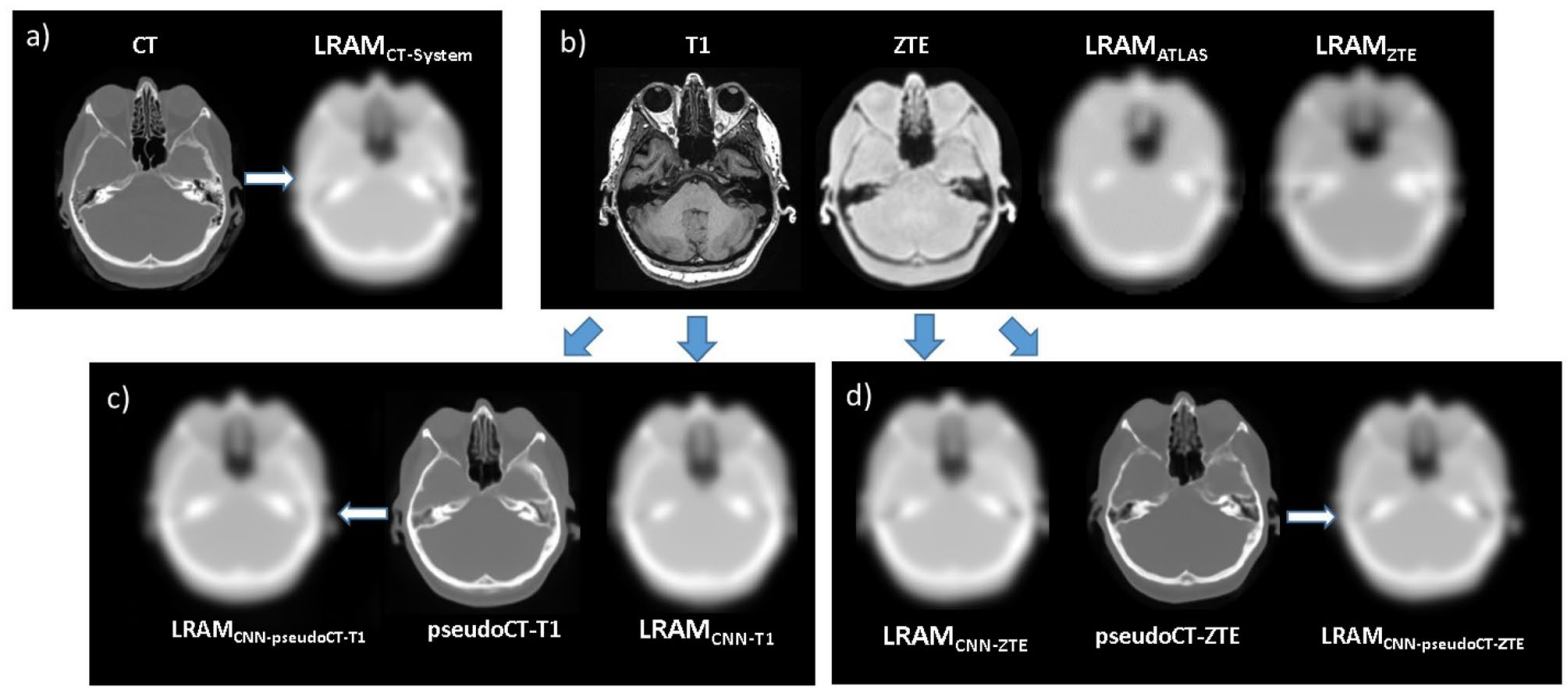
Representative images for a FDG patient: **a** original CT and LRAM_CT-System_, ground truth for AC; **b** MR T1, MR ZTE and LRAM_ATLAS_, LRAM_ZTE_ generated by the PET/MR system for AC; **c** T1-based attenuation maps generated by the UNet: LRAM_CNN-T1_ and pseudoCT-T1, from which LRAM_CNN-pseudoCT-T1_ is obtained; **d** ZTE-based attenuation maps generated by the UNet: LRAM_CNN-ZTE_ and pseudoCT-ZTE, from which LRAM_CNN-pseudoCT-ZTE_ is obtained

Corresponding images are shown in Fig. 2.

CT, LRAM_CT-System-HighRes_ and LRAM_CT-System_ to be given as reference outputs to UNets were obtained by removing the HH. ZTE and T1 volumes to be given as inputs to UNets were obtained by correcting ZTE and T1 volumes for magnetic field inhomogeneity using the N4 algorithm implemented in the 3D Slicer Software [20, 21], by normalizing intensities on a case by case basis by median tissue values and by registering the resulting volumes to corresponding CTs by means of a rigid transform with bilinear interpolation using MIPAV software (https://mipav.cit.nih.gov/). HH was lastly added to volumes estimated by UNets.

### METH PET/MR and CT Data Acquisition

The PET/MR acquisition protocol for METH studies consisted in axial 3D LAVA Flex and 3D ZTE sequences (already described in “FDG PET/MR Data Acquisition”) and in a set of diagnostic MR sequences acquired simultaneously to a 20-min PET scan. Patients had also a diagnostic CT scan (120 kVps, 250–330 mA) performed to exclude complications immediately after surgery, about 1 month before the PET/MR session.

### 511 KeV LRAM Generation for METH patients

METH PET data acquired with the PET/RM scanner were reconstructed using five LRAM volumes:

- LRAM_CT-System_: LRAM generated off line with the Recon Research Tool provided by GEMS (PETTOOLBOX 2.0) starting from the diagnostic CT scan rigidly registered to corresponding RMs with MIPAV software (https://mipav.cit.nih.gov/)
- LRAM_ATLAS_ and LRAM_ZTE_, automatically generated by the SIGNA PET/MR system with atlas-based and segmentation-based methods
- LRAM_CNN-ZTE_: LRAM obtained with a UNet trained on LRAM_CT-System-HighRes_ and ZTE pairs of the sixteen FDG patients, when receiving in input the patient ZTE volume
- LRAM_CNN-pseudoCT-ZTE_: LRAM obtained applying the AC pipeline to pseudoCTs obtained with a UNet trained on CT and ZTE pairs of the sixteen FDG patients, when receiving in input the patient ZTE volume

### PET Reconstruction

FDG PET images were reconstructed with 3D Ordered Subsets Expectation Maximization (OSEM). OSEM parameters were constrained to be consistent to those used in our institution to reconstruct the PET database needed for Statistical Parametric Mapping (SPM) analysis [19] (2 iterations, 24 subsets, FOV 25 cm, voxel grid 128, voxel size 1.95 × 1.95 × 3.27 mm^3^, Transaxial Gaussian Filter of 4 mm FWHM, Axial-Post Filter Standard). METH PET images were reconstructed with 3D OSEM with time of flight (TOF) information. OSEM parameters were: 3 iterations, 16 subsets, FOV 25 cm, voxel grid 128, voxel size 1.95 × 1.95 × 3.27 mm^3^, Transaxial Gaussian Filter of 3 mm FWHM, Axial-Post Filter Standard.

### U-Net Architecture, Implementation and Parameters

**Fig. 3:**
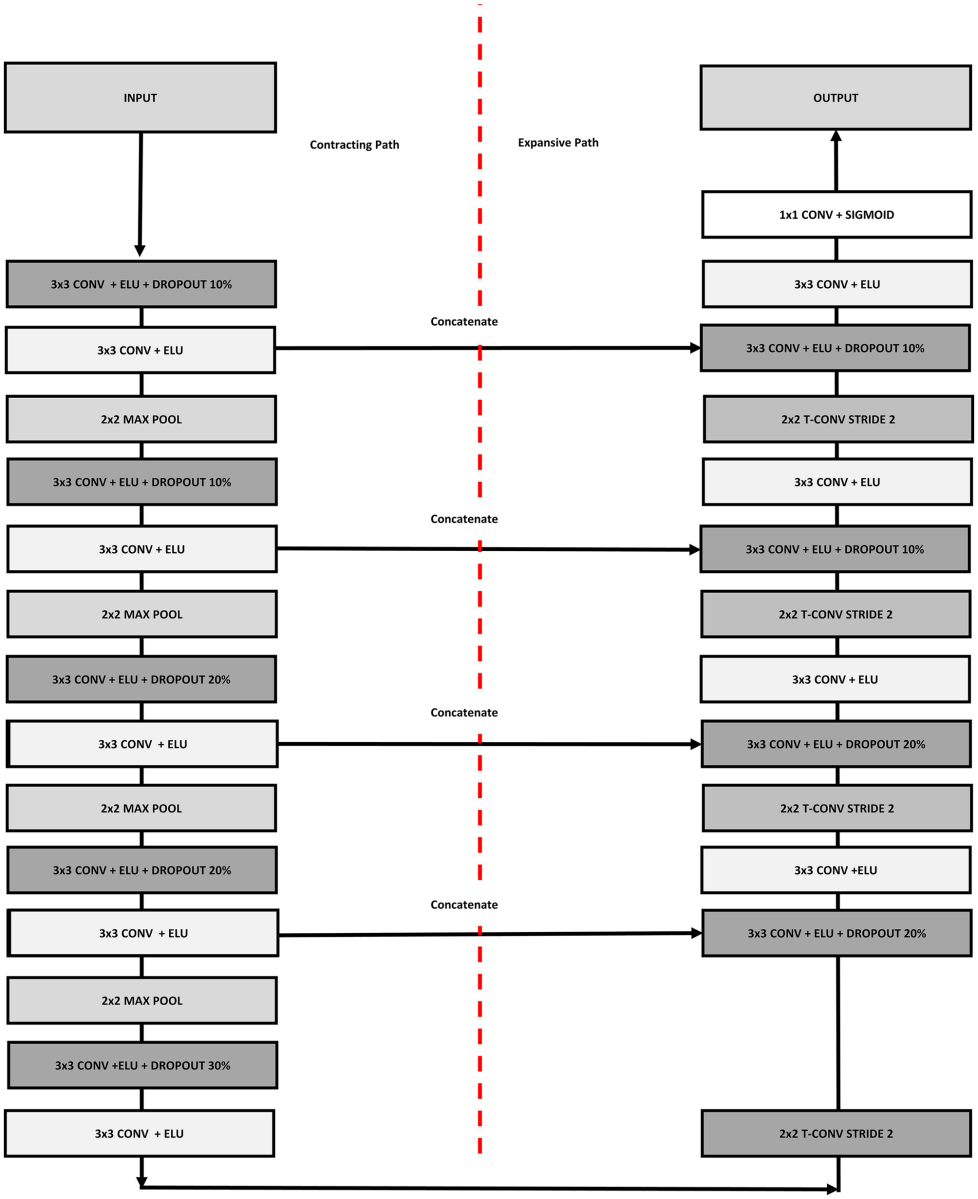
UNet architecture scheme. The same architecture was used in all the experiments

A 2D UNet model was implemented in Keras with TensorFlow as backend on a NVIDIA Quadro RTX 8000 GPU. The network architecture is shown in Fig. 3. The network takes in input a 2D axial slice of the ZTE (T1) volume and provides in output the corresponding slice of the estimated CT or LRAM volume. The UNet is composed by 5 convolution layers in the encoding/decoding part with 3 × 3 kernels and Elu activation function. In encoding, each convolution layer is constituted by a first convolution extracting 16/32/64/128/256 features, by dropout, by a second convolution extracting 16/32/64/128/256 features and by 2 × 2 max-pooling. In decoding, feature maps from deeper layers are deconvolved with stride 2, concatenated with corresponding feature maps coming from the encoding part and given in input to a convolution layer identical to the encoding one. The squared L2 norm of the difference between reference and UNet outputs was used as loss function during training with the Adam stochastic optimization method (learning rate = 0.0005, *β*_1_ = 0.9, *β*_2_ = 0.999). UNet weights were initialized with the He Normal Keras initialization, i.e. weights were sampled from a truncated normal distribution *N*(0,*σ*) with $\sigma =\sqrt{2/\nu }$, where *ν* is the number of input units in the weight tensor.

On the FDG dataset, a leave-one-out (LOO) scheme was used to fairly evaluate the strategy performance on all the sixteen subjects. For each LOO run, twelve of the remaining patients were used for training and three for validation, with an early stopping on validation loss to prevent overfitting. On the METH dataset, UNets trained on the whole FDG dataset were used.

### Data Analysis

As for the FDG dataset, PET images reconstructed with LRAM_CT-system_, LRAM_ATLAS_, LRAM_ZTE_, LRAM_CNN-ZTE_, LRAM_CNN-T1_, LRAM_CNN-pseudoCT-ZTE_, LRAM_CNN-pseudoCT-T1_ and LRAM_CNN-ZTE-Coarse_ were all normalized to the stereotactic space by using the SPM software (https://www.fil.ion.ucl.ac.uk/spm/). PET images were then qualitatively evaluated by two expert nuclear medicine physicians, who looked for differences in the radiotracer distribution depiction and ultimately in the clinical interpretation.

For each patient, a mask was defined on the reference (i.e. PET reconstructed with LRAM_CT-system_) by selecting voxels with more than 1/10 of the FOV mean activity. This mask practically discards all background (air) voxels, which are not of interest for the analysis as they are not impacted by attenuation. PET images were then compared to the reference inside the mask in terms of mean-error (ME) and root mean-square-error (RMSE), defined as follows:

$$M{E}_{PET}=\frac{1}{{\sum }_{{i\in N}_{vox}}{PET}_{REF}}{\sum\limits_{i\in {N_{vox}}}}\frac{PET-{PET}_{REF}}{{N}_{vox}}$$

$$RMS{E}_{PET}=\frac{1}{{\sum }_{{i\in N}_{vox}}{PET}_{REF}}\sqrt{{\sum\limits_{{i\in N}_{vox}}}\frac{{\left(PET-{PET}_{REF}\right)}^{2}}{{N}_{vox}}}$$

**Fig. 4:**
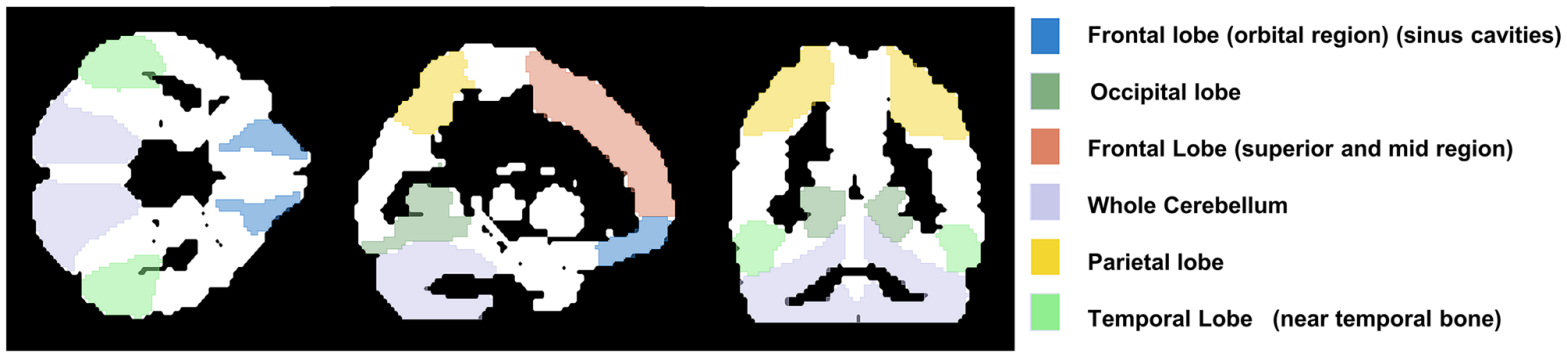
VOIs for regional quantification of FDG PET images

PET images were finally quantified on a regional basis inside six macro volumes of interest (VOIs) (Fig. 4) identified on the automated-anatomical-labelling (AAL) atlas [22]. In each lobe, we specifically chose the most critical areas for attenuation factor estimation: (1) frontal superior orbital and frontal mid orbital region in the frontal lobe; (2) frontal superior and frontal mid region in the frontal lobe; (3) calcarine, cuneus and lingual region in the occipital lobe; (4) parietal superior and inferior areas in the parietal lobe; (5) temporal inferior areas in the temporal lobe; (6) whole cerebellum. Bilateral regions were always used. Significant differences with respect to the reference were assessed by means of the Mann Whitney test.

**Fig. 5:**
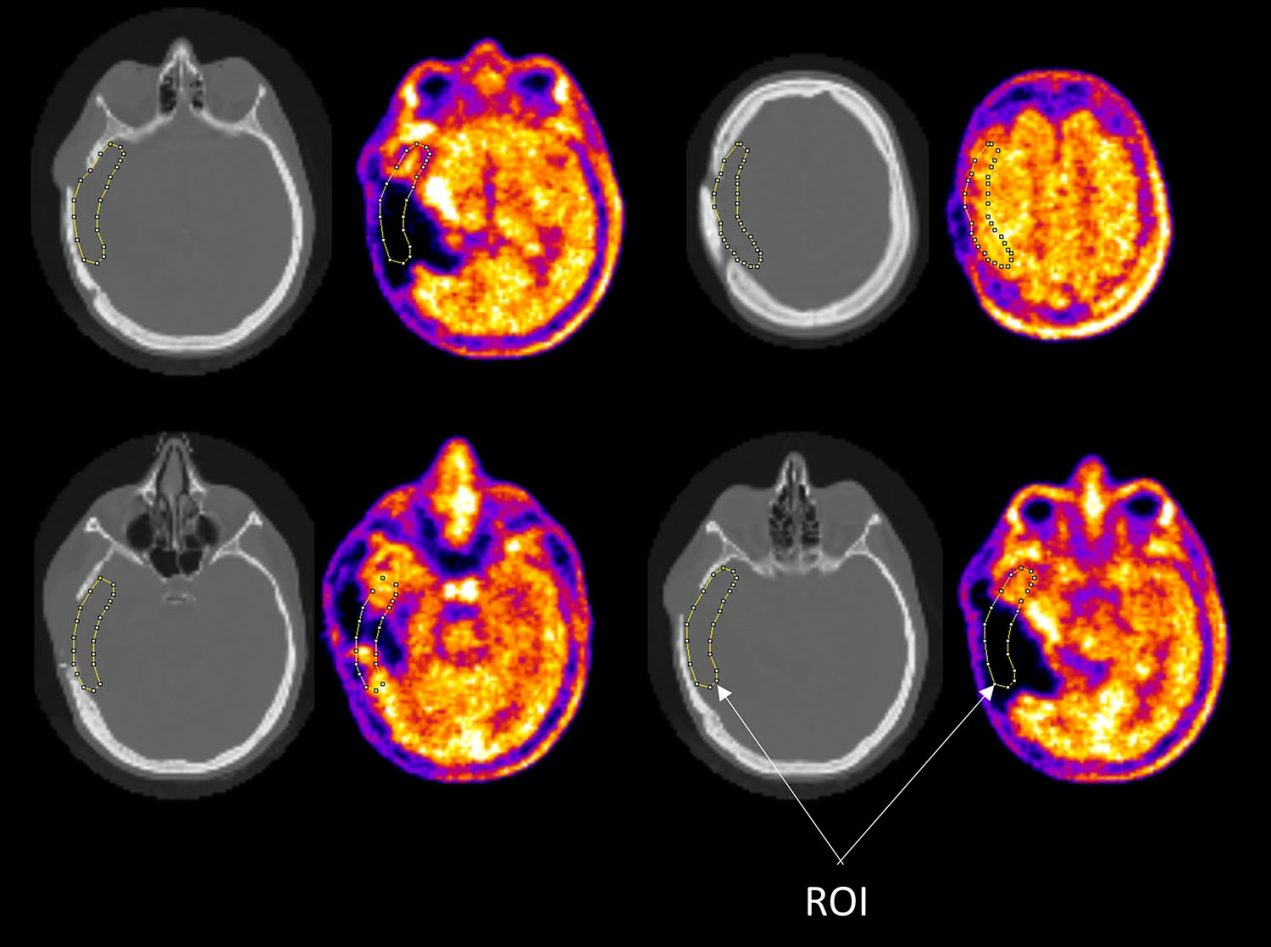
Four representative images (CT (left) and PET (right)) of a METH patient with superimposed ROIs for PET quantification in the anatomically altered region

As for the METH dataset, multiple 2D ROIs were manually delineated on registered CT images in the anatomically altered region. Mean PET counts in each ROI were then computed and compared, taking as reference the counts obtained on PET images reconstructed with LRAM_CT-system_. ROI examples are shown in Fig. 5.

## Results

**Fig. 6:**
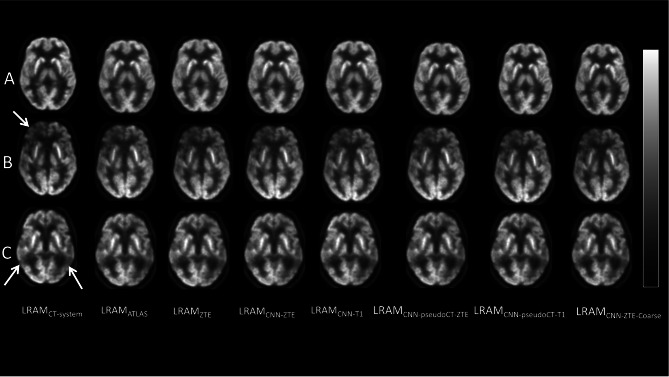
A representative PET transaxial image for three different patients (**A**, **B**, **C**) reconstructed using the eight LRAMs. No visible technical artefacts can be recognized in any of the reconstructed images as well as in the comparison with the reference (first column). Regions of lower uptake (arrows) were confirmed by physicians as result of the patient pathology

As for the FDG dataset, visual image analysis did not reveal any qualitative differences or visually recognizable artefacts in any of the sixteen patients. In Fig. 6, a representative transaxial PET image is shown for three patients (A, B, C) reconstructed using the eight considered LRAMs. Patients B and C show regions of reduced uptake (white arrows) which are due to their brain dysfunction.

**Fig. 7:**
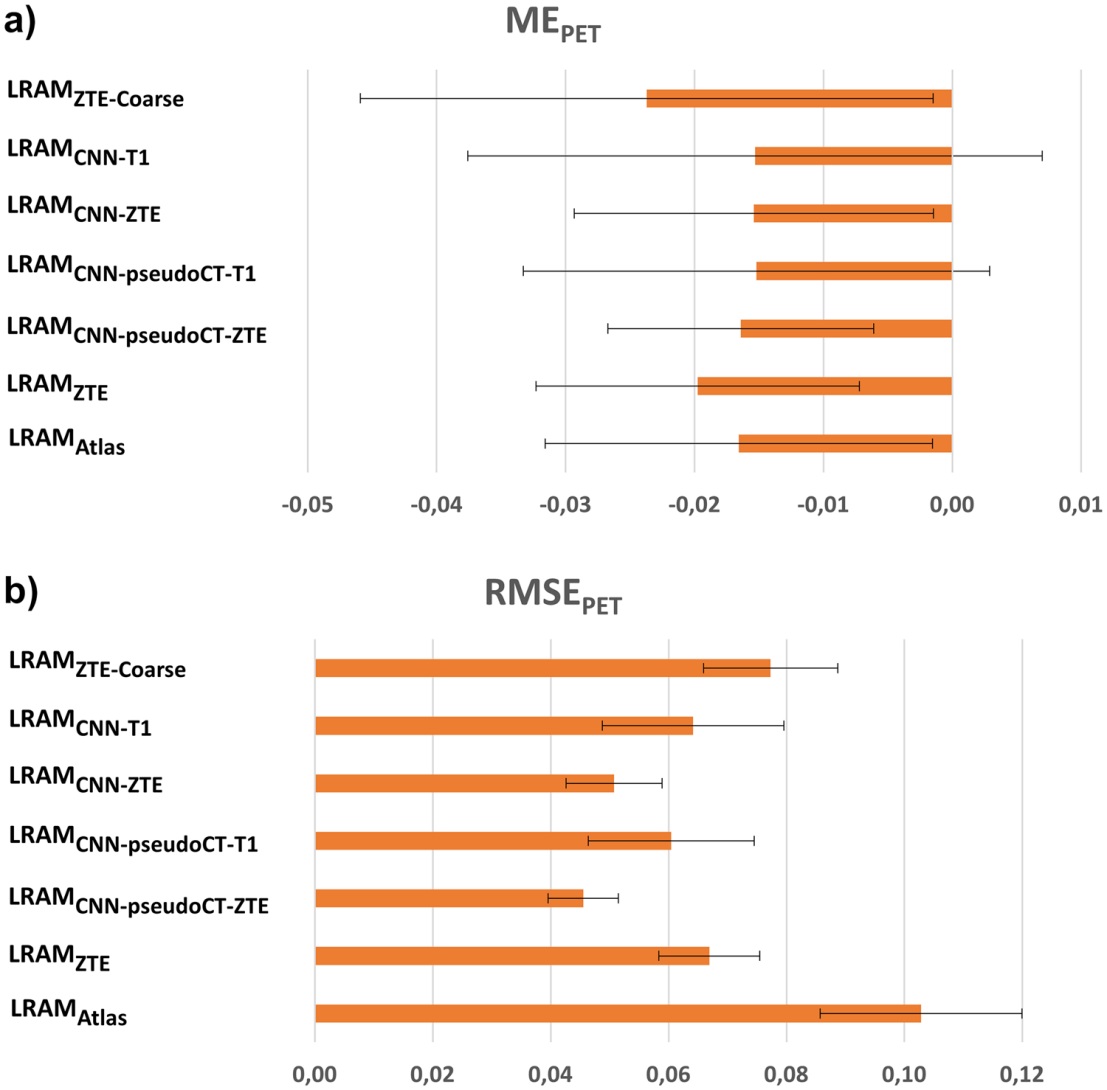
ME and RMSE computed on FDG PET images. For each LRAMs, mean and standard deviation were computed across the sixteen patients

ME and RMSE computed on PET images are shown in Fig. 7.

Atlas-based and segmentation-based LRAMs together with LRAMs computed on the coarse voxel grid provide the largest error on PET images. Errors are smaller and comparable on PET images corrected with LRAMs computed on the finer grid and with LRAMs derived from pseudoCTs. In particular, LRAMs obtained from ZTE provide smaller bias and smaller variability than LRAMs obtained from T1-weighted images.

**Fig. 8:**
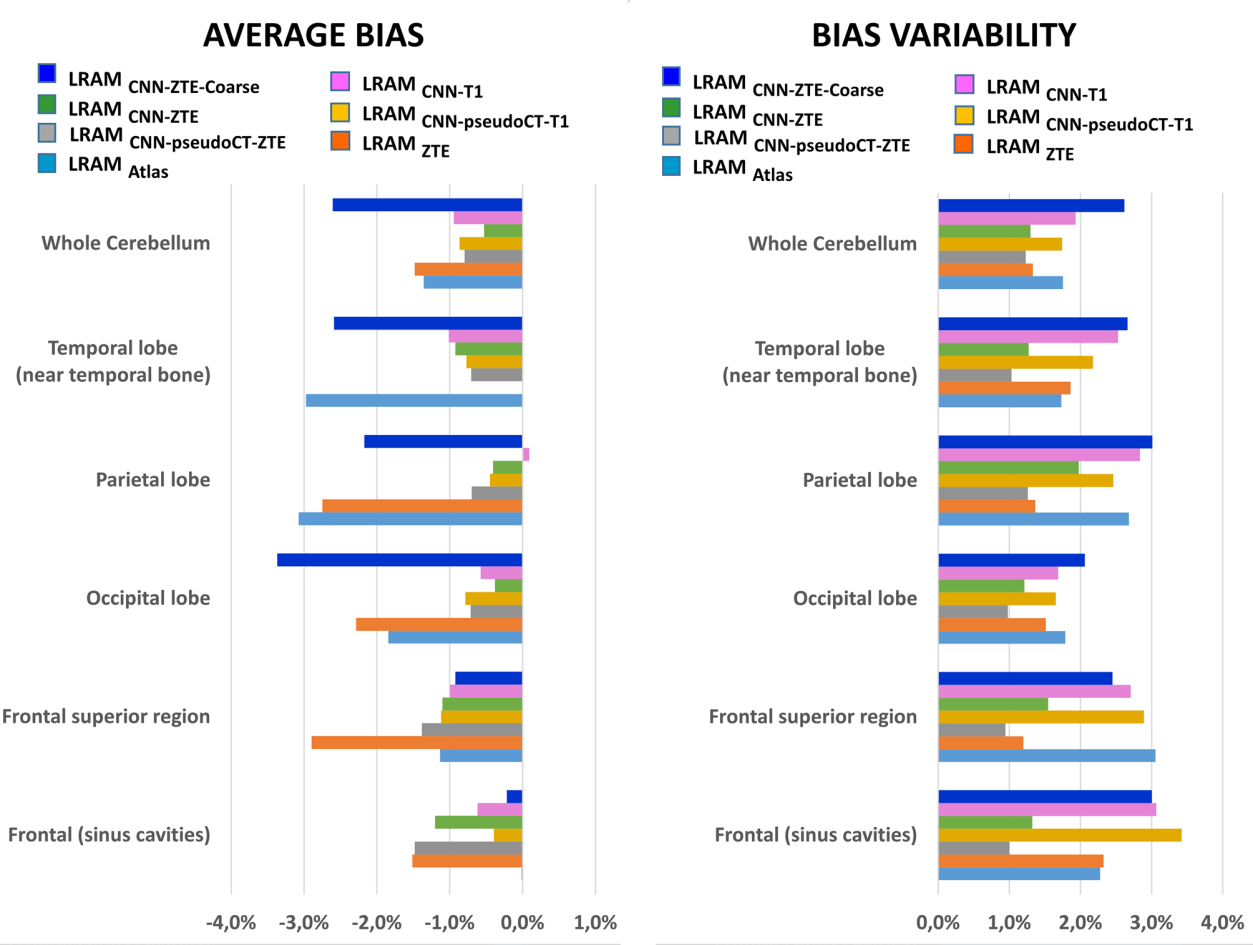
Results of the regional analysis of FDG PET images inside six macro-VOIs

Results of the regional analysis are shown in Fig. 8.

Atlas-based LRAM and segmentation-based LRAM provide a null intersubject 18F-FDG uptake average bias respectively in frontal region and temporal lobe, but larger bias in the other regions. In particular, LRAM_ATLAS_ induces a 3% average bias in temporal and parietal lobes, LRAM_ZTE_ a nearly 3% average bias in parietal lobe and frontal superior region. Similarly, LRAM computed from coarse grid ZTE generates a negligible average bias in the frontal region, but an unacceptably large bias in most of the other regions. LRAMs derived from pseudoCT and those directly estimated on the finer grid, from both ZTE and T1-weighted images, perform more regularly among regions, with an intersubject 18F-FDG uptake average bias always smaller than 1.5%. The intersubject bias variability is large for LRAM_CNN-ZTE-Coarse_ and for LRAMs generated starting from T1-weighted images. It is instead the lowest (always lower than 2%) for LRAMs obtained with UNet and ZTE images. No differences with respect to the reference resulted statistically significant.

**Fig. 9:**
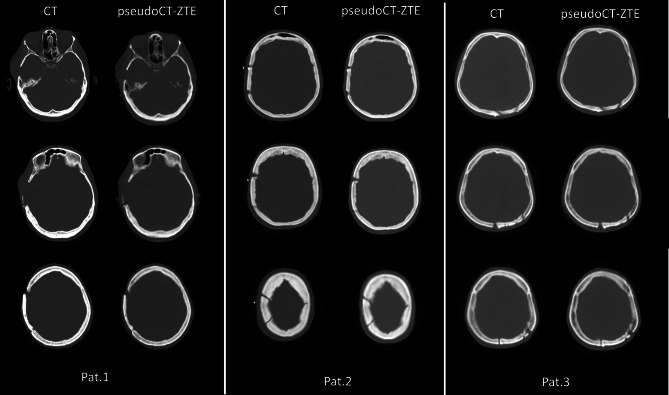
METH patients. Original CTs and corresponding pseudoCTs generated by the UNet

As for the METH dataset, original CTs and corresponding pseudoCTs generated by the UNet trained on the anatomically normal FDG patients are shown in Fig. 9. Anatomic variations appear all well reconstructed.

**Fig. 10:**
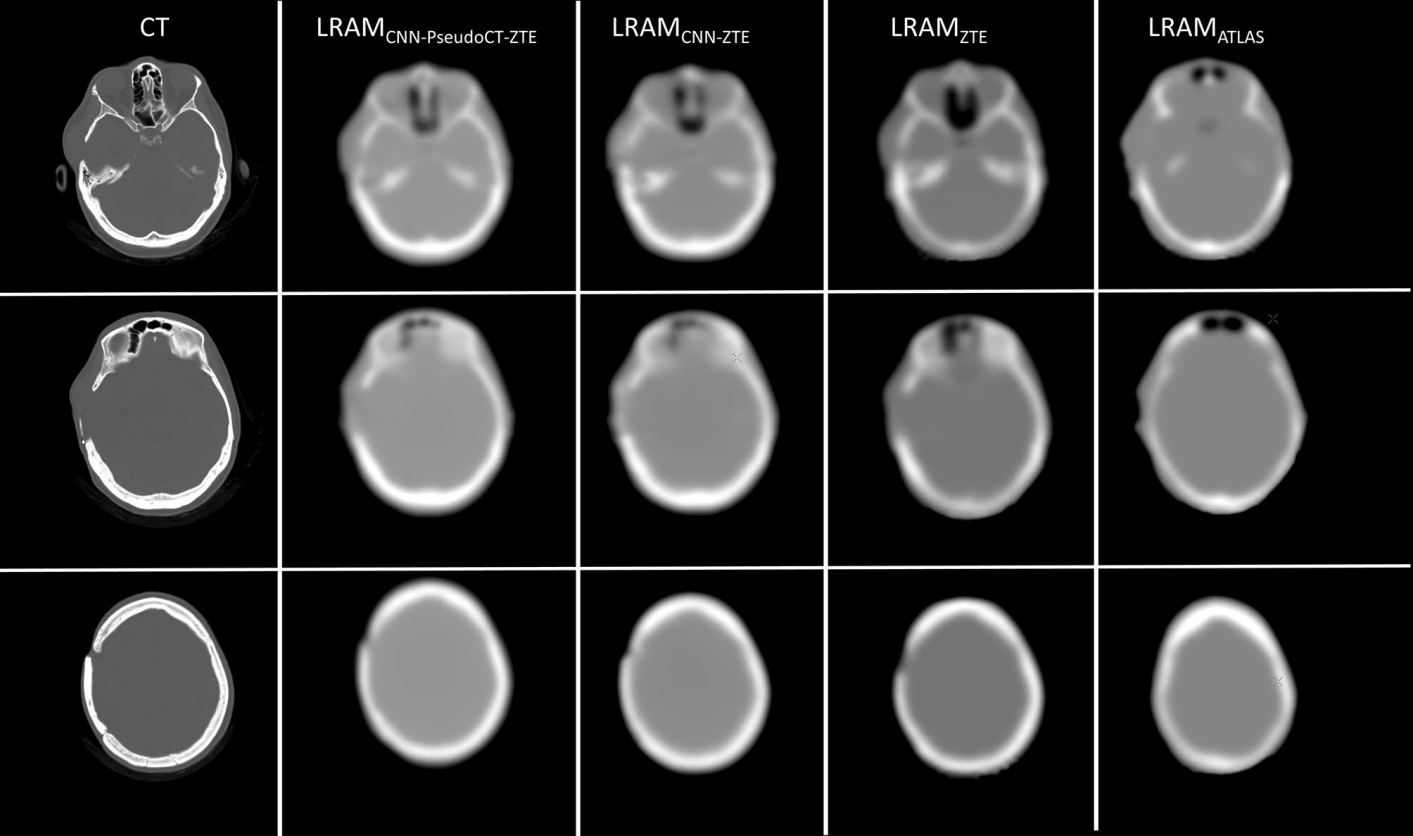
Three representative sections of a METH patient: original CT (left) and corresponding LRAMs, LRAM_CNN-pseudoCT-ZTE_, LRAM_CNN-ZTE_, LRAM_ZTE_, LRAM_ATLAS_

In Fig. 10, original CTs and LRAMs generated by the PET/MR system (LRAM_ZTE_, LRAM_ATLAS_) and by the UNet (LRAM_CNN-pseudoCT-ZTE_, LRAM_CNN-ZTE_) are shown for a METH patient.

**Fig. 11:**
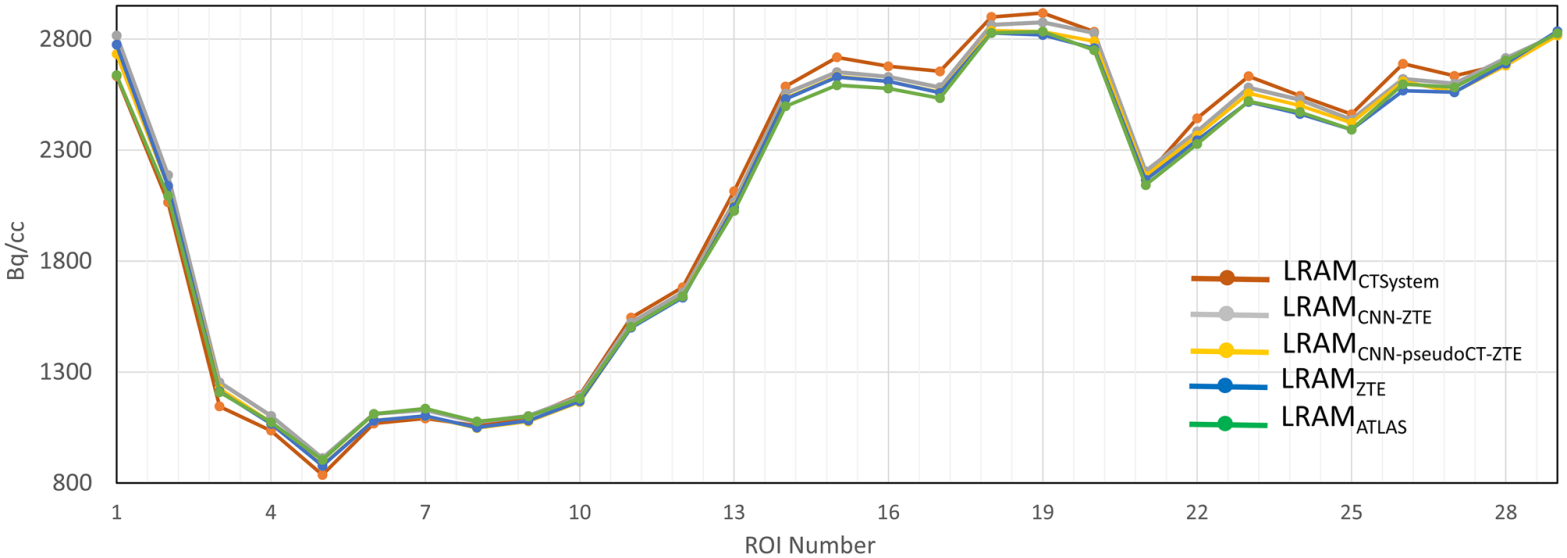
Results of the regional analysis for a METH patient (over 29 ROIs). Mean counts are computed on PET images reconstructed with different LRAMs inside ROIs manually delineated on cranial anatomical abnormalities

Results of the regional analysis for a METH patient (over 29 ROIs corresponding to 29 brain consecutive image sections) are shown in Fig. 11. LRAM_CNN-ZTE_ and LRAM_CNN-PseudoCT-ZTE_ appear able to provide values similar to LRAM_CT-System_ with respect to LRAM_ZTE_ and LRAM_ALTAS_. Globally, LRAM_CNN-ZTE_, LRAM_CNN-PseudoCT-ZTE_ and LRAM_ZTE_ obtained a mean error smaller than 2% in all the three patients; LRAM_ATLAs_ on a patient got a mean error of 4%.

## Discussion

Many works in literature have already shown that deep learning strategies applied to MR images in PET/MR are able to provide synthetic AC maps, and consequently PET images, more accurate than segmentation or atlas-registration strategies. In this work, we investigated the best MR image to be used and the best point of the AC pipeline to insert the synthetic map in. For this reason, we used the same UNet architecture throughout the study and evaluated all the possible inputs/outputs combinations.

The assessment was firstly done on FDG PET data acquired on a PET/CT scanner and reconstructed with the vendor offline tool. MR images of the same patient to be given in input to the UNet were acquired on a PET/MR scanner. MR images were corrected for bias field inhomogeneity, normalized and registered to CT images; MR coils were removed and substituted with the CT HH. We can confirm the results obtained by other authors [2] about the greater importance of data pre-processing (bias field correction and spatial registration) compared to CNN architecture parameters and hyperparameters (results not shown). We think that, probably, even better results could be obtained by further improving these data pre-processing steps.

Sixteen patients were considered and a LOO training/testing scheme was adopted. A 2D UNet with 5 levels of convolution in the encoding/decoding parts was successfully trained on 12 patients with an early stopping on the validation loss computed on 3 patients. PET images reconstructed with AC maps estimated by the UNet were quantitatively compared to reference PET reconstructed with AC maps derived from acquired CT data. Attenuation maps generated by the PET/MR scanner were also assessed, for comparison.

Results show that UNet AC models generally provide an estimate of 18F-FDG uptake with a lower bias than state-of-the-art atlas-based or segmentation-based AC methods. This confirms the results previously obtained by other authors [4, 8, 12–14]. As for the best point in the AC pipeline to insert the synthetic map, results show that it is nearly equivalent to generate synthetic CT or directly LRAM after bilinear scaling and low pass filtering. Synthetic CTs provide a mean average bias of −0.7% (−1.0%) and a mean average variability of 2.4% (1.1%) among brain regions, if obtained from T1-weighted (ZTE) images; LRAM has a mean average bias of −0.7% (−0.8%) and a mean average variability of 2.5% (1.4%). It is instead not recommended to generate LRAM after the coarse resampling: in this condition, average bias and bias variability nearly double. The MR image resampling on the coarse grid induces in fact an excessive detail loss which leads to the reconstruction of incorrect LRAM and PET images. As regards instead the best MR sequence, results show that the average bias among patients is nearly the same on images corrected with AC maps obtained from ZTE and T1-weighted images. Only in the frontal region the average bias is larger on images coming from ZTE images (about −1.3%) and lower on images coming from T1-weighted images (about −0.5%). If, however, we look at the bias variability, results clearly show that the least variability is obtained with AC methods that exploit ZTE information. This also confirms the results previously obtained by other authors [16]. In particular, we observed that the same UNet architecture for CT (LRAM) generation provides on average a 120% (70%) lower variability when working on ZTE images than on T1 images. This can be explained by the fact that a CNN can always “learn” the characteristics of the population on average, but accurate prediction at the individual level requires subject-specific information to be present in the input layer. Overall, we can conclude that UNet models working on ZTE and generating synthetic CT or LRAM provide the best results in terms of both accuracy and variability.

To contextualize these numbers within the clinical application, we analysed a typical ^18^F-FDG brain analysis pipeline, which involves pixel-wise statistical comparisons against a pool of healthy controls [23]. We found that, after image scaling to the mean brain activity, the coefficient of variation among subjects lies between 7 and 9%, depending on the location. This means that, even at very liberal statistical thresholds (*p* < 0.01; uncorrected), regions are highlighted as hypometabolic only when the activity reduction is at least larger than 14%. Indeed, in a group comparison on a large number of Alzheimer’s disease subjects [23], we observed a 19% average activity reduction in the most impacted region, the parietal lobe. It is therefore very important to have a systematic bias less than 1% in all the regions and a standard deviation smaller than 2% as provided by ZTE-based AC methods, since these values are mostly guaranteed not to significantly influence the outcome and therefore to provide robust quantification.

We successively assessed the UNet ability to provide AC maps on three PET/MR METH patients with anatomical abnormalities due to surgery. We did not use T1 images in this case, but only ZTE images which intrinsically contain the whole information needed for AC. UNets trained on the sixteen anatomically normal FDG patients on ZTE/CT and on ZTE/LRAM_CT-System_ pairs resulted able to correctly reconstruct bone abnormalities, with improved accuracy with respect to LRAM_ATLAS_ and LRAM_ZTE_. The generation of LRAM volumes, which intrinsically have a low resolution, resulted less sensitive to such anatomical variations respect to the generation of high resolution CT volumes. PET mages were quantified near bone abnormalities and results were compared with those obtained with AC coming from coregistered diagnostic CTs. Results confirmed those obtained on the FDG dataset. Further studies on this kind of patient/lesions are however needed.

This study has several limitations, which will be discussed in the following.

- We chose to use a 2D UNet instead of the best-performing 2.5D or 3D UNets because of the scarce number of patients. However, we do not believe that the results obtained in terms of best MR sequence and best synthetic image insertion point would change as the network changes. Other strategies have been recently proposed in literature, such as GAN or cycleGAN [13], which could provide better results thanks to their robustness to misregistration. The generator used by most of this network is however a 2D UNet, like the one used in this study. Furthermore, these networks require a larger number of patients to be trained. We think we should invest in these networks for abdominal PET/MR AC, where misregistration is definitely a challenge; in brain studies, simpler strategies may be sufficient.
- In this work, the AC correction pipeline implemented on our PET/CT and PET/MR scanner was considered. Other vendors probably use slightly different pipelines, e.g. with a reduced low pass filter FWHM of with a less coarse resampling grid. Indeed, this was one of the reasons motivating our choice to compare synthetic image insertion into different points of the AC pipeline. If nearly equivalent PET was obtained generating CTs or LRAMs after 10 mm filtering, we think that equivalent PETs will be also obtained if generating less filtered LRAMs.
- A further limitation is due to the smaller axial FOV of the PET/CT scanner (15.7 cm) compared to the wider axial FOV of the PET/MR scanner (25.0 cm). In order to obtain pairs of well spatially correlated CT-MR data, we necessarily had to remove the lower neck region from MR images. However, considering that the lower neck region is generally not crucial for brain neurological evaluations, this limitation reasonably seems not critical for the work.

## Conclusions

In this work, we generated synthetic images for AC correction in brain PET/MR from ZTE or T1 images by means of a 2D UNet. The first objective was to understand which of the two MR sequences is able to provide more accurate AC maps and PET images. Results obtained on the 16 anatomically normal FDG patients show that ZTE provides a comparable intersubject average bias on PET images, but a lower intersubject bias variability with respect to T1. ZTE results appear also accurate on the 3 methionine patients containing cranial anatomy abnormalities due to surgery. Well knowing the PET/MR AC pipeline which does not make a full usage of the synthetic CT information but uses a reduced contrast and a reduced spatial resolution version (LRAM), as a second objective, we wanted to understand whether there are differences in generating UNet images at different points of the AC pipeline. Results obtained on both the 16 anatomically normal FDG patients and the 3 methionine patients show that you can equivalently generate synthetic CTs or LRAMs after bilinear scaling and smoothing but before coarse resampling.

## Data Availability

Data are available upon reasonable request.
